# Pulmonary Adenoleiomyomatous Hamartoma: Case Report of a Rare Entity With Comprehensive Literature Review

**DOI:** 10.1155/crip/6894556

**Published:** 2026-05-22

**Authors:** Hebatullah Elsafy, Alykhan S. Nagji, Ameer Hamza

**Affiliations:** ^1^ Pathology and Laboratory Medicine, Kansas University Medical Center, Kansas City, Kansas, USA, kumc.edu; ^2^ Cardiovascular and Thoracic Surgery, Kansas University Medical Center, Kansas City, Kansas, USA, kumc.edu

**Keywords:** adenoleiomyomatous, hamartoma, lung, pulmonary hamartoma

## Abstract

Pulmonary adenoleiomyomatous hamartomas represent a rare and intriguing entity in pulmonary pathology. This study presents a unique case of adenoleiomyomatous hamartoma along with a comprehensive analysis of 14 cases identified through a systematic review of the literature. A 69‐year‐old Caucasian female presented for evaluation of an incidentally discovered, PET nonavid and slow‐growing pleural‐based nodule in the medial aspect of the lower lobe of her right lung. The biopsy showed pulmonary parenchyma with chronic inflammation, fibrosis, and smooth muscle hyperplasia. Subsequently, a diagnosis of pulmonary adenoleiomyomatous hamartoma was made on wedge resection after the exclusion of differential diagnoses. The literature review suggests a mean age of 54.5 ± 3.5 years at diagnosis and male predominance with a male‐to‐female ratio of 6:1. Follow‐up data on our patient and literature suggest a uniformly benign course. The key takeaways include the indolent radiologic growth pattern. From a pathologic standpoint, excluding mimics such as solitary fibrous tumor, inflammatory myofibroblastic tumor, PEComa, Langerhans cell histiocytosis, mesothelial proliferations, and IgG4‐related diseases is crucial.

## 1. Introduction

Pulmonary hamartoma is the most prevalent benign tumor of the lung [1, 2]. The incidence of pulmonary hamartoma in the general population is around 0.25%, exhibiting a skewed male‐to‐female ratio ranging from 2:1 to 4:1, with a median age of manifestation at 60 years [1]. Clinically, a pulmonary hamartoma is typically an asymptomatic entity, frequently discovered incidentally during imaging procedures conducted for unrelated medical concerns [2]. Radiologically, it usually manifests as a peripheral nodule characterized by rounded margins. Pathologically, it is composed predominantly of mature mesenchymal tissues, notably cartilage and adipose tissue [3]. The World Health Organization (WHO) classification categorizes pulmonary hamartomas as benign tumors composed of mature tissues native to the site of origin, including mesenchymal, epithelial, and mixed elements [4]. These are further subclassified based on their predominant tissue component, such as chondroid, lipomatous, myomatous, or adenomatous, among others. This classification underscores the heterogeneous nature of these lesions, encompassing various histological subtypes and presentations [5–8].

Within the spectrum of noncartilaginous pulmonary hamartomas, adenoleiomyomatous hamartoma is an exceedingly rare entity, with only 14 documented cases in the literature.

In this report, we present a unique case of adenoleiomyomatous hamartoma in a Caucasian female. This presentation is noteworthy given the rarity of adenoleiomyomatous hamartomas, particularly in females, and the typical predilection for left‐sided lesions in the literature. Furthermore, we aim to enrich the literature by conducting a systematic review, incorporating the 14 documented cases of adenoleiomyomatous hamartomas to provide a comprehensive understanding of this rare entity.

## 2. Case Report

**Figure 1:**
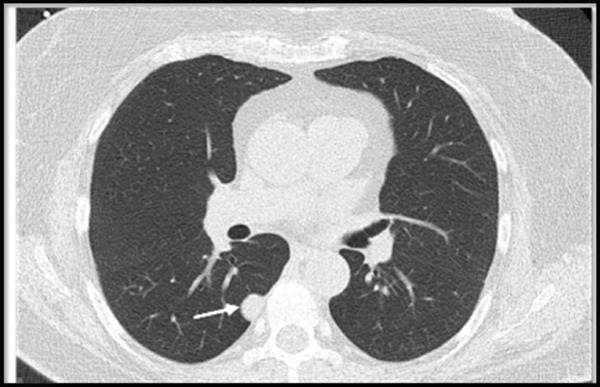
CT scan demonstrating a well‐demarcated homogenous noncalcified nodule measuring 1.8 × 1.6 cm.

**Figure 2:**
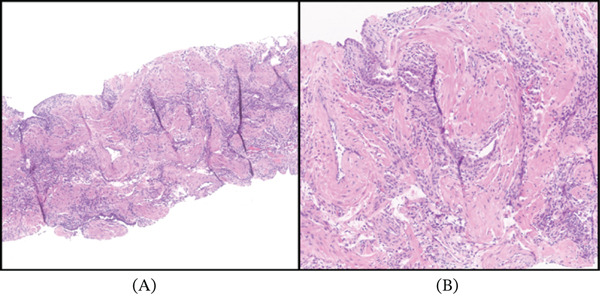
(A) Low‐power photomicrograph of the biopsy specimen showing smooth muscle fibers and chronic inflammatory infiltrate (H&E 50x). (B) Medium power showing smooth muscles with entrapped epithelial component (H&E 100x).

**Figure 3:**
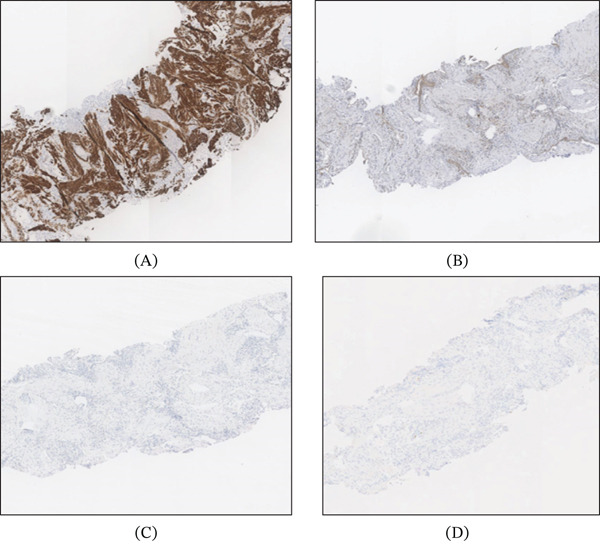
(A) Staining for desmin IHC stain (desmin 50x). Negativity for (B) STAT6 (STAT6 50x), (C) ALK (ALK 50x), and (D) IgG4 stains (IgG4 50x).

A 69‐year‐old Caucasian female presented for the evaluation of an incidentally discovered pleural‐based nodule in the medial aspect of the lower lobe of her right lung. The nodule was discovered at an outside institution in 2016 and measured 1.1 cm at that time. A CT chest performed at our institution in 2022 showed the nodule to be 1.7 cm, and a repeat CT in 2023 confirmed a 1.8 cm nodule (Figure 1) with a growth rate of essentially 1 mm/year. Pulmonary function testing showed normal FVC, FEV1, FEV1/FVC ratio, and FEF 25%–75%. The patient had no history of cancer, smoking, occupational or industrial inhalational exposures, or asbestos exposure. A positron emission tomography (PET)/CT examination showed a nonavid nodule with low uptake of 18F‐fluorodeoxyglucose. The biopsy was performed, which showed pulmonary parenchyma with chronic inflammation, fibrosis, and smooth muscle hyperplasia (Figure 2). Immunohistochemical (IHC) stains for desmin and SMA were positive only focally, highlighting the smooth muscle hyperplasia and excluding a smooth muscle scar or a leiomyoma (Figure 3), while stains for ALK, HMB45, CD34, STAT6, and CD1a were all negative, rendering the entities, including inflammatory myofibroblastic tumor (IMFT), PEComatous lung lesions, solitary fibrous tumor (SFT), and Langerhans cell histiocytosis (LCH), less likely. Subsequently, a wedge resection was performed. No intraoperative frozen section evaluation was performed.

### 2.1. Pathological Findings of the Wedge Resection

In the wedge resection specimen, the tumor, measured 1.8 × 1.7 × 1.4 cm, exhibited a firm, solid consistency and appeared homogenous white in color upon sectioning. No other lesions were identified within the grossly normal adjacent lung parenchyma.

### 2.2. Histological Examination

**Figure 4:**
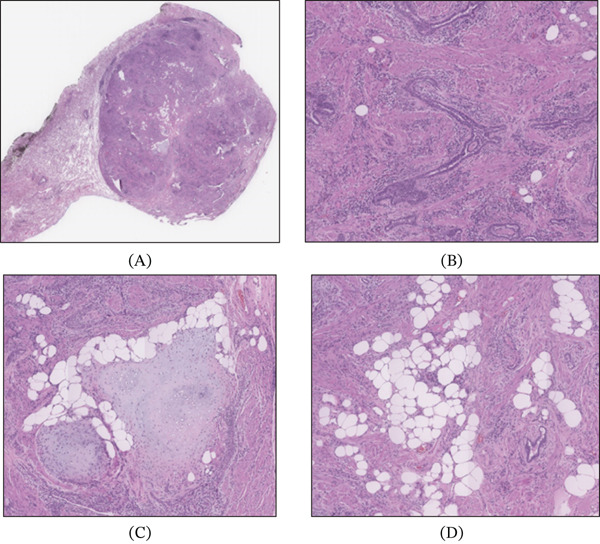
(A) Wholemount demonstrating a well‐circumscribed tumor (H&E 5x). Medium photomicrographs from different areas showing the (B) smooth muscle hyperplasia with entrapped benign epithelial elements (H&E 50x) and (C) focal areas of cartilaginous (H&E 50x) and (D) adipocytic differentiation (H&E 50x).

Histologically (Figure 3), the tumor was well‐demarcated and composed of entrapped benign epithelium and mesenchymal components. The mesenchymal component displayed irregular fascicles and whorls of smooth muscle fibers and fibroblasts. Focal areas of cartilaginous and adipocytic differentiation were also noted (Figure 4). There were no areas of cellular atypia or polymorphism, increased mitosis, or necrosis.

**Figure 5:**
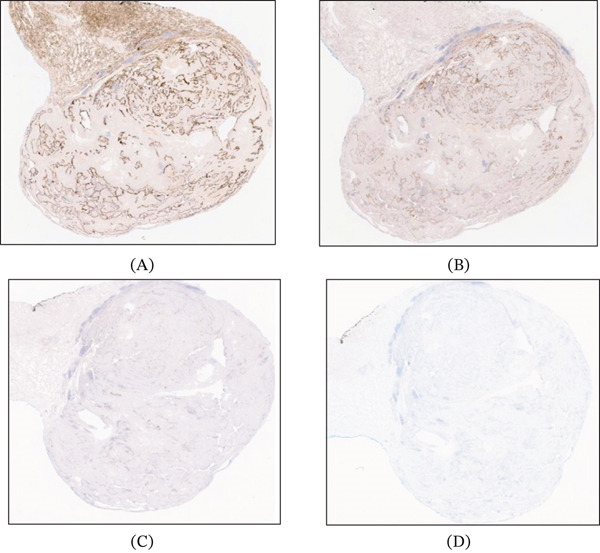
Staining of epithelial component for (A) EMA (EMA 5x) and (B) TTF1 (TTF1 5x) stains. Negativity for (C) CD1a (CD1a 5x) and (D) HMB45 (HMB45 5x) stains.

IHC analysis revealed the epithelial component to be positive for epithelial membrane antigen (EMA) and thyroid transcription factor‐1 (TTF1) (Figure 5). The mesenchymal component exhibited positivity for desmin, while HMB45, CD1a, and calretinin were negative. IgG4 and CD138 stains showed no evidence of IgG4‐related disease.

The final diagnosis of adenoleiomyomatous hamartoma was rendered. The patient is alive, well, and disease‐free 21 months after surgery.

## 3. Literature Review

PubMed and Scopus databases were searched from inception to December 2024. The Medical Subject Headings (MeSH) terms used included (lung/hamartoma [MeSH terms]) OR (adenoleiomyomatous hamartoma) AND (fibroadenomyomatous hamartoma) OR (mucinous adenoleiomyomatous hamartoma). No restrictions on article type or publication date were used, and 86 results were retrieved. This was followed by a manual search of the references. After removal of duplicated articles and articles without access to full‐text reports, a total of 14 reported cases were identified. These cases are summarized in Table 1 [9–17].

**Table 1:** Summary of cases from the literature review.

Case and ref.	Age	Gender	Smoking Hx	Location	Size (cm)	Diagnosis	IHC	Genetic	Prognosis
1 [9]	55	M	Yes	LUL	0.2	Fibroleiomyomatous hamartoma	Epithelial: TTF1(+); SPA(+); KRT(+); p53(−); Ki‐67(0.2%) Mesenchymal: VIM(+); SMA(+); HHF35(+); CD34(−); p53(−); Ki‐67(0.3%); PGR(+)	No mutation in KRAS and EGFR	AW (4 months)
2 [10]	53	M	NA	RUL	0.4	Fibroleiomyomatous hamartoma	N/A	No mutation in KRAS and EGFR	AW (12 months)
3 [11]	54	M	NA	LLL	1.5	Fibroleiomyomatous hamartoma	Epithelial: SPA(+); KRT(+); CC10(+) Mesenchymal: SMA[+]; desmin(+); PGR(−); EGR(−); AGR(−); Ki‐67(0.1%)	NA	NA
4 [12]	53	M	NA	LLL	2.5	Adenomyomatous hamartoma	Epithelial: KRT(+) Mesenchymal: VIM(+); SMA(+); desmin(+); CD34(−); S100(+); EGR(−); PGR(−); p53(−); Ki‐67(0.3%)	NA	NA
5 [13]	69	M	NA	NA	NA	Fibroleiomyomatous hamartoma	NA	NA	NA
6 [14]	59	M	Yes	LLL	1.1	Adenomyomatous hamartoma	NA	NA	AW (16 months)
7 [15]	76	M	NA	LLL	1.8	Adenomyomatous hamartoma	Epithelial: Three types of glands (mucinous: TTF1(−) and KRT20(+) and small glands and epithelial tufts: TTF1(+)), the three types CD34(−) and CDX2(−). Mesenchymal: (desmin(+), SMA(+), calponin(+)). Ki‐67 low proliferation index in all components.	NA	NA
8 [16]	60	F	Never	LLL	0.9	Mucinous adenomyomatous hamartoma	Epithelial: Entrapped bronchioles (TTF1(+) and P63(+)), oncocytic metaplastic cells and serous cells (lactoferrin(+) and lysozyme(+)) Mesenchymal: Desmin(+) Ki‐67 low proliferation index in all components.	NA	NA
9 [17]	58	M	Former	LLL	1.7	Mucinous adenomyomatous hamartoma	Epithelial: TTF1(−), napsin(−), CK7 and pan‐CK (+), MUC1,2,6(−), and MUC5AC (+) Mesenchymal: Desmin(+), caldesmon(+), and SMA(+)	Wild‐type ALK, ROS1, and BRAF	AW (24 months)
10 [17]	60	M	Former	LLL	2.1	Mucinous adenomyomatous hamartoma	Epithelial: TTF1(−), napsin(−), CK7 and pan‐CK(+), MUC1,2,6(−), and MUC5AC(+) Mesenchymal: Desmin(+), caldesmon (+), SMA (+)	Wild‐type ALK, ROS1, and BRAF	AW (7 years)
11 [17]	72	M	Never	LUL	5.5	Mucinous adenomyomatous hamartoma	Epithelial: TTF1(−), napsin(−), CK7 and pan‐CK(+), MUC1,2,6(−), and MUC5AC(+) Mesenchymal: Desmin(+), caldesmon(+), and SMA(+)	Wild‐type ALK, ROS1, and BRAF	AW (39 months)
12 [17]	67	M	Former	RUL	3.5	Mucinous adenomyomatous hamartoma	Epithelial: TTF1(−), napsin(−), CK7 and pan‐CK(+), MUC1,2,6(−), and MUC5AC (+) Mesenchymal: Desmin(+), caldesmon(+), and SMA(+)	Wild‐type ALK, ROS1, and BRAF	NA
13 [17]	59	F	NA	LLL	2	Mucinous adenomyomatous hamartoma	Epithelial: TTF1(−), napsin(−), CK7 and pan‐CK(+), MUC1,2,6(−), and MUC5AC(+) Mesenchymal: Desmin(+), caldesmon(+), and SMA(+)	Wild‐type ALK, ROS1, and BRAF	AW (3 years)
14 [17]	49	M	NA	RLL	3	Mucinous adenomyomatous hamartoma	Epithelial: TTF1(−), napsin(−), CK7 and pan‐CK (+), MUC1,2,6(−), and MUC5AC(+) Mesenchymal: Desmin(+), caldesmon(+), and SMA(+)	Wild‐type ALK, ROS1, and BRAF	AW (41 months)
15 (present)	69	F	Never	RLL	2.1	Adenomyomatous hamartoma	Epithelial: TTF1(+) and EMA (+) Mesenchymal: Desmin(+), SMA(+), ALK(−), HMB45(−), and STAT6(−)	NA	AW (15 months)

*Note:* Size, centimeters; HHF35, muscle‐specific actin.

Abbreviations: AGR, androgen receptor; ALK, anaplastic lymphoma kinase; AW, alive and well; CC10, Clara cell secretory protein; CK7, cytokeratin 7; EGR, estrogen receptor; F, female; HMB45, human melanoma black; KRT, cytokeratin; LLL, left lower lobe; LUL, left upper lobe; M, male; MUC, mucicarmine; NA, not available; pan‐CK, pancytokeratin; PGR, progesterone receptor; RLL, right lower lobe; RUL, right upper lobe; SMA, *α*‐smooth muscle actin; SPA, surfactant apoprotein A; STAT6, signal transducer and activator of transcription; TTF1, thyroid transcription factor‐1; VIM, vimentin.

## 4. Discussion

Adenoleiomyomatous hamartoma of the lung is a rare benign tumor that often presents as an incidental finding on imaging studies or during surgical procedures. Despite its benign nature, its rarity and varied clinical presentations make it an interesting entity for clinicians and pathologists alike.

This uncommon variant of pulmonary hamartoma has been described using various diagnostic terms, including fibroleiomyomatous hamartoma, adenomyomatous hamartoma, or adenofibroma. It is characterized by exuberant smooth muscle fascicles and an entrapped tubular or cleft‐like epithelial component and is either completely devoid of other mesenchymal elements or demonstrates only a minor component thereof.

Demographically, adenoleiomyomatous hamartomas of the lung occur in a middle‐aged to older adult population. Analysis of the 14 reported cases shows an age range from 49 to 60 years, with a mean of 54.5 ± 3.5 and male predominance with a male‐to‐female ratio of 6:1. Our patient was female, 62 years old at initial presentation and 69 at the time of surgery.

Shifting the focus to smoking status, an analysis of the 14 cases revealed varying trends. Among the 14 patients, two individuals were current smokers, three were former smokers, and two had never smoked. For the remaining cases, smoking history was not specified. Our patient was a nonsmoker.

Examining the location of lesions, the majority of the 14 cases displayed involvement in the lower left lobe (eight cases), followed by the left upper lobe (two cases), right upper lobe (two cases), and right lower lobe (one case). In our patient, the lesion was in the right lower lobe.

Radiologically, these lesions may manifest as solitary pulmonary nodules or masses with variable calcification patterns, including the so‐called popcorn calcification, with variable enhancement patterns on contrast‐enhanced imaging. Although PET‐CT was performed in our patient and demonstrated a nonavid nodule, prior reports rarely comment on PET‐CT characteristics. Tao et al. [8] described slightly increased FDG uptake in their case. Overall, adenoleiomyomatous hamartomas may demonstrate low metabolic activity on PET‐CT, aligning with their benign nature.

Regarding the size of the lesion, the review uncovered a range spanning from 0.2 to 5.5 cm with a mean of 2.7 ± 1.7. Our case fits within this spectrum, with the lesion measuring 1.8 cm.

Microscopically, the tumor in our patient was well‐demarcated and composed of a mixture of entrapped benign epithelium and mesenchymal components. The mesenchymal component exhibited irregular fascicles and interwoven bundles of smooth muscle fibers and fibroblasts, forming a fibromyomatous architecture. In addition, focal areas of cartilaginous and adipocyte differentiation were observed. Notably, there were no areas of severe atypia, cellular polymorphism, increased mitotic activity, or necrosis, supporting the benign nature of the lesion.

Upon reviewing the 14 previously reported cases with similar histological features, a spectrum of related entities was identified. Four cases were described as fibroleiomyomatous hamartomas, where the fibrous and smooth muscle components predominated, while three were termed adenomyomatous hamartomas, characterized by a dominant smooth muscle component with entrapped epithelial structures. Seven cases were classified as mucinous adenomyomatous hamartomas, in which mucin production was the additional defining feature. Our case, however, differs in that mucin was entirely absent. While the mesenchymal component in our case exhibited both fibroblastic and smooth muscle differentiation, it also demonstrated focal cartilaginous and adipocyte elements, which were not consistently reported in all previously described cases.

Turning to IHC staining, a noteworthy comparison arises with the TTF1 stain. Among the nine cases subjected to this IHC analysis, three cases expressed TTF1, while six cases were negative. Notably, the latter subset was predominantly associated with the diagnosis of mucinous adenoleiomyomatous hamartoma [14]. Our case had a TTF1‐positive epithelial component, aligning with the literature, as our case did not have mucinous features.

Furthermore, regarding keratin staining, our case, along with 11 cases reported in the literature, demonstrated positive staining. Ki‐67 IHC staining was performed in only four cases, with all exhibiting a low proliferation index. Regarding desmin and SMA staining, a consistent pattern of positive staining was expected and noted in 11 cases, mirroring the findings in our case.

Perez‐Atayde and Seiler [15], in an electron microscopy study, suggest that the histogenesis of the epithelial components of the lesion was actually a result of entrapment by expanding mesenchymal components. This conclusion was based on the presence of intact basement membranes surrounding the epithelial nests and well‐formed cell junctions, demonstrating these glands were pre‐existing rather than true neoplastic components.

From a pathologic standpoint, the most vital task is to rule out other possible differential diagnoses, especially in a small biopsy specimen. Some of these include SFT, IMFT, perivascular epithelioid cell tumor (PEComa), LCH, mesothelial proliferations, and IgG4‐related disease, each of which has distinct histologic and IHC characteristics.

SFTs are well‐circumscribed neoplasms composed of spindle cells arranged in a haphazard fashion with staghorn‐like vascularity. Their IHC profile is characterized by strong nuclear STAT6 positivity, along with CD34 positivity, while SMA and desmin are typically negative [16].

IMFTs exhibit spindle cell proliferation with a prominent lymphoplasmacytic infiltrate and varying degrees of fibrosis. These tumors frequently express ALK, SMA, and desmin but are usually negative for CD34 [17].

PEComas demonstrate a distinct perivascular growth pattern and are composed of epithelioid to spindle cells with clear or granular cytoplasm. They frequently express melanocytic markers such as HMB45 and Melan‐A, along with frequent positivity for SMA and desmin, helping to distinguish them from other mesenchymal tumors [18, 19].

LCH is characterized by grooved, coffee bean–shaped nuclei with eosinophilic infiltrates and is identified immunohistochemically by CD1a and Langerin (CD207) positivity [20].

Mesothelial proliferations, including benign and malignant mesotheliomas, can sometimes mimic mesenchymal lung tumors, particularly when there is spindle cell morphology. However, these lesions are typically positive for calretinin, WT1, and D2‐40, which help differentiate them from other spindle cell neoplasms.

Lastly, IgG4‐related disease can present with storiform fibrosis and dense lymphoplasmacytic infiltrates, often raising concern for an inflammatory or fibrotic tumor. The hallmark of IgG4‐related disease is a high IgG4‐positive plasma cell count, confirmed by IgG4 IHC staining [21].

Given the broad differential diagnosis, careful morphologic assessment and a targeted IHC panel are necessary to exclude these entities. In our case, morphology, along with the lack of STAT6 expression, excluded SFT; the absence of ALK expression decreased the likelihood of IMFT; and negative HMB45 and Melan‐A staining made PEComa unlikely. Additionally, negative CD1a and Langerin staining excluded LCH, while calretinin negativity reduced the likelihood of mesothelial proliferation. There was no significant IgG4 plasma cell infiltrate to suggest IgG4‐related disease.

It is prudent to clarify here that, whereas this panel of IHC stains made the entities in the differential diagnosis less likely, it is insufficient to completely exclude them on its own. Whereas the majority of the IMFTs harbor an ALK alteration that can be detected immunohistochemically, there is a significant number of tumors with alterations in other receptor tyrosine kinase proteins in which the ALK stain would be negative. In case of PEComas, while they frequently express both myoid and melanocytic markers, they do not always express markers of both lineages, and the markers they do express can vary considerably. Mesothelial proliferations are typically positive for calretinin, WT1, and D2‐40; however, some mesothelial tumors, especially spindle cell mesothelioma, can lose expression of any or all of these markers. Essentially, nothing is 100% in medicine. In the end, the clinical picture, radiologic findings, morphology, and IHC stains are all parts of the puzzle and play a key role in arriving at the right diagnosis.

Recent investigations into pulmonary hamartomas have shed light on their molecular underpinnings. High occurrences of rearrangements involving 6p21 or 12q14‐15 have been documented [22]. Combining IHC with cytogenetics revealed translocations at 6p21 in the mesenchymal components, while expression of HMGI‐C and HMGI(Y) proteins, due to rearrangements at 6p21 and 12q15, respectively, was predominantly noted in the nuclei of mesenchymal components [23, 24]. These findings bolster the proposition that neoplastic mesenchymal proliferation characterizes pulmonary hamartomas, driven by abnormal gene expression resulting from HMGI‐C or HMGI(Y) rearrangements. However, there is no evidence suggesting the neoplastic nature of the epithelial components.

Despite the diagnostic challenges when unusual features are present and associated with adenoleiomyomatous hamartomas, the prognosis of this condition remains favorable. Our analysis of reported outcomes, including the eight cases with available follow‐up data, reveals a uniformly benign course, with all patients reported to be alive and well. This favorable prognosis underscores the nonaggressive nature of adenoleiomyomatous hamartomas and emphasizes the importance of accurate diagnosis and appropriate management strategies in optimizing patient outcomes.

## Funding

No funding was received for this manuscript.

## Conflicts of Interest

The authors declare no conflicts of interest.

## Data Availability

The data that support the findings of this study are available from the corresponding author upon reasonable request.
